# Comprehensive health risk analysis of heavy metal pollution using water quality indices and Monte Carlo simulation in R software

**DOI:** 10.1038/s41598-023-43161-3

**Published:** 2023-09-22

**Authors:** Ahmad Badeenezhad, Hamed Soleimani, Samaneh Shahsavani, Iman Parseh, Amin Mohammadpour, Omid Azadbakht, Parviz Javanmardi, Hossein Faraji, Kamal Babakrpur Nalosi

**Affiliations:** 1 Department of Environmental Health Engineering, School of Medical Sciences, Behbahan Faculty of Medical Sciences, Behbahan, Iran; 2 Department of Environmental Health Engineering, School of Public Health, Tehran University of Medical Sciences, Tehran, Iran; 3 Student’s Scientific Research Center, Tehran University of Medical Sciences, Tehran, Iran; 4 Department of Environmental Health Engineering, Research Center for Health Sciences, Institute of Health, Shiraz University of Medical Sciences, Shiraz, Iran; 5 Department of Environmental Health Engineering, School of Health, Student Research Committee, Shiraz University of Medical Sciences, Shiraz, Iran; 6 Department of Radiobiology and Radiation Protection, Behbahan Faculty of Medical Sciences, Behbahan, Iran; 7 Department of Environmental Health Engineering, Ahvaz Jundishapour University of Medical Sciences, Ahvaz, Iran; 8 Health Systems Research, Health Research Institute, Babol University of Medical Sciences, Babol, Iran

**Keywords:** Environmental sciences, Environmental social sciences, Diseases, Health care, Risk factors

## Abstract

Rapid urbanization, population growth, agricultural practices, and industrial activities have led to widespread groundwater contamination. This study evaluated heavy metal contamination in residential drinking water in Shiraz, Iran (2021). The analysis involved 80 groundwater samples collected across wet and dry seasons. Water quality was comprehensively assessed using several indices, including the heavy metals evaluation index (HEI), heavy metal pollution index (HPI), contamination degree (CD), and metal index (MI). Carcinogenic and non-carcinogenic risk assessments were conducted using deterministic and probabilistic approaches for exposed populations. In the non-carcinogenic risk assessment, the chronic daily intake (CDI), hazard quotient (HQ), and hazard index (HI) are employed. The precision of risk assessment was bolstered through the utilization of Monte Carlo simulation, executed using the R software platform. Based on the results, in both wet and dry seasons, Zinc (Zn) consistently demonstrates the highest mean concentration, followed by Manganese (Mn) and Chromium (Cr). During the wet and dry seasons, 25% and 40% of the regions exhibited high CD, respectively. According to non-carcinogenic risk assessment, Cr presents the highest CDI and HQ in children and adults, followed by Mn, As and HI values, indicating elevated risk for children. The highest carcinogenic risk was for Cr in adults, while the lowest was for Cd in children. The sensitivity analysis found that heavy metal concentration and ingestion rate significantly impact both carcinogenic and non-carcinogenic risks. These findings provide critical insights for shaping policy and allocating resources towards effectively managing heavy metal contamination in residential drinking water.

## Introduction

Groundwater, a life-sustaining source, is imperilled as an unwitting repository of trace elements that wield formidable health risks. Heavy metals can infiltrate our bodies through contaminated water, air, and food, posing a threat to human health. This situation emerges from rapid urbanization, population growth, intensified agriculture, and industrial activities, surpassing natural processes and human actions^1–3^. The gravity of the situation is noteworthy, as toxic heavy metal exposure can lead to neurological disorders, cancers, and even fatalities. This extends beyond individuals, affecting plants that unwittingly enter the food chain, escalating risks. Amidst this complexity, understanding diverse heavy metals and their health effects is crucial for deciphering contaminated groundwater hazards^4,5^.

For instance, arsenic exposure has been linked to skin lesions, cardiovascular diseases, and cancer^6,7^. Zinc, although essential, can cause gastrointestinal disturbances^8^. Lead exposure, especially in children, can lead to developmental delays, neurological disorders, and cognitive impairments^9^ Cadmium is linked to kidney damage, lung diseases, and cancer risk. Chromium exposure harms respiration, increasing lung cancer risk. Copper toxicity causes gastrointestinal symptoms, liver damage, and impaired kidney function. Elevated Manganese levels associate with neurotoxicity, cognitive issues, and movement disorders^10^. Reliable water quality indices and health risk assessment methods are essential for understanding the potential health risks associated with heavy metal concentrations.

Water quality indices are pivotal in assessing contamination levels and potential risks linked to heavy metals within water sources^11^. They offer a structured framework to evaluate overall water quality and the extent of heavy metal pollution. Amid the diverse array of water quality indices, several hold notable significance in the context of heavy metal contamination assessment. Among these is the heavy metals index (MI), a parameter considering the concentrations of distinct heavy metals within water samples^12^. MI is precisely calculated to assess water resource drinkability. Heavy metal pollution index (HPI) is another way to estimate water quality based on heavy metals and their effect on human health^13^. The heavy metals evaluation index (HEI) also connects heavy metal concentrations and toxicity levels, offering insights into potential health risks^14^. The contamination degree (Cd) evaluation model determines the combined effects of several qualitative parameters, which can affect drinking water quality unfavourably^13,15^.

Following the discussion on water quality indices, the subsequent focus shifts to the methodology for assessing risks associated with heavy metals. Deterministic health risk assessment utilizes fixed values and assumptions to estimate risks, considering exposure pathways, toxicological data, and population characteristics. On the other hand, probabilistic approaches consider uncertainties and variations in exposure and toxicity data, providing a more comprehensive evaluation of potential risks^16^. The probabilistic approach employed in Monte Carlo simulation (MCS) enhances the accuracy and reliability of health risk assessments by accounting for the variability and uncertainty in input parameters. Monte Carlo simulation (MCS) analysis is a powerful and widely used method for assessing health risks associated with heavy metal contamination in water. Unlike traditional deterministic approaches, MCS considers the variability and uncertainty in input parameters, providing a more comprehensive and realistic estimation of health risks^17^.

This study introduces a novel approach by integrating water quality indices (HEI, HPI, Cd, and MI), deterministic and probabilistic (Monte Carlo simulation) methods for carcinogenic and non-carcinogenic health risk assessment in Shiraz drinking water, Iran. Additionally, the study leverages the power of R software for conducting Monte Carlo simulations. R software plays a pivotal role as it enables researchers to execute complex simulations with high precision and efficiency. Its extensive libraries and statistical capabilities are crucial in handling the variability and uncertainty in input parameters, providing a more comprehensive and realistic estimation of health risks associated with heavy metal contamination^18^. This integration represents a significant advancement in evaluating health risks associated with heavy metal contamination, offering a more holistic perspective than previous studies. By applying these advanced techniques, the research provides updated and in-depth insights into heavy metal pollution in the area under investigation.

## Materials and methods

### Study area

**Figure 1:**
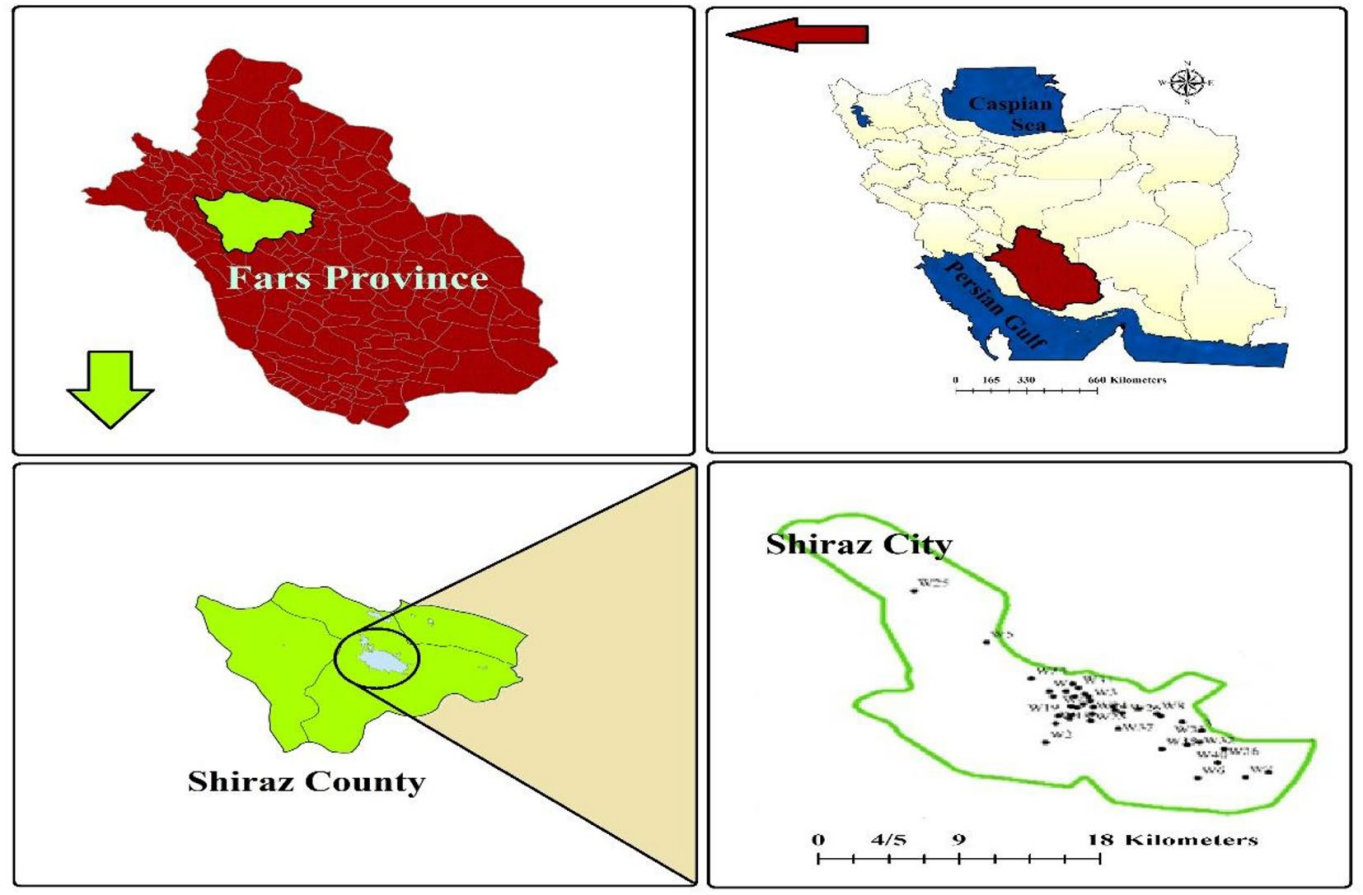
Location of the sampling site: Shiraz City, Iran.

The study area was Shiraz City, located in the Fars province of southwestern Iran. The city covers an area of 1268 square kilometers and has a rectangular shape with a length of approximately 40 km and a width ranging from 15 to 30 km. Shiraz is the fifth-largest metropolis in Iran, with a population exceeding 1,565,572, according to the 2016 census report. It is the capital of Fars province and is situated in the picturesque Zagros mountain range. Shiraz is located at 29° 36ʹ 37ʹʹ N and 52° 31ʹ 52ʹʹ E. This city is 1486 m above sea level and experiences an average annual rainfall of 337 mm. In the warmest month of the year, July, Shiraz experiences an average temperature of 30 °C, while in the coldest month, January, the average temperature drops to 5 °C. During April, the temperature reaches an average of 17 °C, and in October, it settles at around 20 °C. Overall, the city maintains an average annual temperature of 18 °C. The sampling area’s location is depicted in Fig. 1.

### Water sampling and analysis

In this cross-sectional study conducted in 2021, we collected 80 water samples during wet and dry seasons from 40 designated stations. These stations were selected based on careful consideration of geographical distribution, proximity to potential contamination sources, and representation of diverse environmental conditions. This deliberate station selection ensures the samples’ representativeness and enhances our findings’ reliability. The collection of samples followed the guidelines outlined in the Standard Methods for Water and Wastewater Examination^19^. Before sampling, the Polypropylene sampling containers were thoroughly washed and cleaned using a diluted nitric acid solution and deionized water. Stagnant water within the pipeline was removed by briefly activating the tap. The sampling points’ precise geographical coordinates were meticulously recorded using a portable GPS device (Model No. GARMIN MONTANA 650)^20^. The quantification of contaminant concentrations in the water samples was performed utilizing graphite furnace atomic absorption spectrometry (Perkin Elmer AA-Analyst 200), a well-established and dependable analytical technique renowned for its accuracy and precision in determining the levels of various contaminants in aqueous samples. This method enables precise and reliable measurements of the concentration levels of contaminants, ensuring robust and accurate data acquisition for the subsequent assessment of water quality^21,22^. Subsequently, the collected water samples were meticulously labelled and stored in a cool box containing ice packs, ensuring a constant temperature of 4 °C, following standard conditions. The samples were then transported to the laboratory for further analysis. Upon arrival at the laboratory, a specific standard solution was prepared to facilitate the evaluation of the concentrations of various heavy metals, including Cd, Pb, Hg, As, Cu, Cr, Zn, Fe, and Mn. The concentrations of these metals were measured and recorded in micrograms per liter (μg/l) using voltammetry techniques (Metrohm 797 V), a reliable analytical technique^23^.

#### Quality control

Before use, all sample bottles underwent a thorough cleaning process involving the washing of the bottles with diluted nitric acid (HNO_3_) followed by rinsing with deionized water. Blank samples were examined after every set of five samples, and this process was iterated three times to ascertain the accuracy and precision of the analytical method utilized. Furthermore, standard reference materials were utilized for each element as a benchmark to assess the accuracy and precision of the concentration analysis of the targeted heavy metals.

### Non-carcinogenic risk assessment

Risk management entails assessing the likelihood and health impacts of incidents caused by environmental risk factors on humans and animals^24^. The study includes an important non-carcinogenic risk assessment to determine the potential health hazards of metals in drinking water. In order to assess the non-carcinogenic risk associated with heavy metals, it is crucial to determine the Chronic Daily Intake (CDI) for each exposure pathway. The CDI values, expressed in milligrams per kilogram per day (mg/kg/day), are calculated for the selected heavy metals considering the ingestion route. Table 1 gives the input parameters in the CDI formula, and Eq. (1) provided below are employed to calculate the CDI values^25^:

1 $$\text{CDI}=\frac{\text{C}\times \text{IR}\times \text{EF}\times \text{ED}}{\text{BW}\times \text{AT}},$$

where *CDI* chronic daily intake (mg/kg/day), *Ci* the individual metal concentrations (μg/l), *IR* the ingestion rate (l/day), *EF* exposure frequency (days/year), *ED* The exposure duration (year), *BW* the average body weight (kg/person), *AT* the average time (in days).

**Table 1:** Values of parameters used in health risk assessment methods in different age groups.

Parameters	Unit	Children	Adult
Ingestion rate (IR.)	l/day	1	2
Exposure frequency (EF.)	Days/year	365	365
Exposure duration (ED.)	Year	6	70
Body weight (BW.)	kg	15	70
Average time (AT)	Days	ED × 365	ED × 365

The estimation of the hazard quotient (HQ) or non-carcinogenic risk value for an individual element involves the utilization of the following mathematical Eq. (2)^26^:

2 $${\text{HQ}} = \frac{{{\text{CDI}}}}{{{\text{RFD}}}},$$

where *HQ* hazard quotient, *RFD* reference dose.

The RfDing or chronic oral reference dose is a parameter utilized to estimate the daily oral exposure level for the human population, including sensitive groups, that is expected to pose minimal risks of harmful effects over a lifetime. The RfDing values for specific elements were determined as follows: For Arsenic (As), the RfDing value was established at 0.0003; for Cadmium (Cd), it was set at 0.0005; for lead (Pb), it is 0.0035, and for Chromium (Cr), it was determined as 0.003 mg/kg/day^27^.

The potential risk to humans from exposure to multiple heavy metals can be assessed using the chronic hazard index (HI). The HI is calculated as the sum of individual hazard quotients (HQs) for each heavy metal. The HQ or HI values indicate the magnitude of non-carcinogenic risks associated with the exposure. The HQ or HI value below one indicates no significant non-carcinogenic risks to human health. However, if the HQ or HI value equals or exceeds one, it signifies significant non-carcinogenic risks, which increase as the HQ or HI value increases. The HI value can be determined using the Eq. (3)^25^:

3 $$HI=\sum HQ,$$

where *HI* hazard index.

The HI provides a comprehensive assessment of the cumulative risk of multiple heavy metals, considering the combined effects of their HQs. By comparing the calculated HI value with the threshold of one, researchers can determine the level of non-carcinogenic risks associated with exposure to the evaluated heavy metals^26^.

### Carcinogenic risk assessment

Toxic metals exposure, even in low concentrations, can cause disorders in the human body (neurological disorders, different types of cancers, and death in acute cases). Long-term consumption of contaminated water with heavy metals increases the danger of cancer in humans. This study conducted carcinogenic risk assessments for a range of heavy metals. The metals that were assessed for their carcinogenic risk include Arsenic (As), Cadmium (Cd), and Chromium (Cr). The categorization of these elements into carcinogens and non-carcinogenic risks is determined according to the guidelines outlined by authoritative organizations such as the United States Environmental Protection Agency (EPA) and the International Agency for Research on Cancer (IARC)^28^. In evaluating the carcinogenic risk, crucial parameters, including the oral reference dose (RfD) and oral slope factor (CSF), are considered for chromium, cadmium, and arsenic, as detailed in Table 2. The carcinogenicity of these elements is assessed by quantifying the Excess Lifetime Cancer Risk (ELCR), which is determined using Eq. (4)^29,30^:

4 $$\text{ELCR}=\text{CDI}\times \text{SF},$$

where *ELCR* excess lifetime cancer risk, *CDI* chronic daily intakes (mg/kg/day), *SF* cancer slope factor (mg/kg/day).

**Table 2:** Standard values of RfD and SF in the current study^7^.

Parameters	Non-carcinogenic	Carcinogenic
	RfD (mg/kg/day)	SF. (mg/kg/day)
As	0.0003	1.5
Cr	0.003	0.19
Cd	0.0005	0.38
Pb	0.0035	–
Zn	0.3	–
Cu	0.04	–
Mn	0.14	–

The calculated ELCR will be compared to the acceptable maximum risk recommended by the United States Environmental Protection Agency (USEPA), which is ≤ 1 × 10^–6^. If the calculated ELCR surpasses this threshold, it indicates a potential health risk to the individuals exposed. Furthermore, the non-carcinogenic risk assessment encompasses lead (Pb), which is not classified as a carcinogen, specifically through the ingestion pathway of drinking water^31^.

### Water contamination indices

#### Metal index (MI)

In evaluating water quality, various indices are employed to assess the level of contamination and the potential risks associated with heavy metals. One of the commonly used indices is the metal index (MI). The MI is a water quality indicator that evaluates the overall contamination level based on the concentrations of various metals compared to their respective maximum allowable concentration (MAC) values. A higher metal concentration concerning its MAC value indicates poorer water quality. If the MI value exceeds 1, it serves as a warning threshold. The MI is calculated using (Eq. 5)^32^:

5 $$\text{MI}=\sum \frac{\text{Ci}}{(\text{M}.\text{A}.\text{C}.)},$$

where *Ci* the concentration of each metal, *MAC* the maximum allowable concentration.

When MI is less than 1, it signifies that the water is suitable for drinking, indicating compliance with safety standards. On the other hand, when MI exceeds 1, it indicates that the water is unsuitable for drinking due to elevated metal concentrations, suggesting potential health risks. The threshold limit of MI equal to 1 serves as a critical danger threshold, highlighting the point at which water quality transitions from drinkable to non-drinkable. This threshold is a significant determinant in assessing the safety and suitability of water for human consumption^33^.

#### Heavy metal pollution index (HPI)

The HPI is an important tool for evaluating heavy metal pollution in water sources. It comprehensively evaluates multiple heavy metals in the water and their collective impact on water quality. The calculation of the HPI involves assigning a weightage factor to each heavy metal based on its toxicity and potential health risks. The weightage factors are determined through extensive scientific research and regulatory guidelines. These factors reflect the relative importance of each metal in contributing to overall pollution and its potentially detrimental effects on human health and the environment. The HPI is typically calculated using the Eq. (6)^34,35^:

6 $$\text{HPI}=\frac{\sum_{\text{i}=1}^{\text{n}}\text{Wi Qi}}{\sum_{\text{i}=1}^{\text{n}}\text{Wi}}.$$

In the given Equation, Wi denotes the unit weightage assigned to the ith parameter (As–Cr, Mn: 0.02, Cd: 0.3, Cu: 0.001, Ni: 0.05, Pb: 0.7, and Zn: 0.0002). Qi represents the sub-index value of the ith parameter, and n represents the total number of parameters considered. The sub-index (Qi) for each parameter is determined using Eq. (7)^36^:

7 $$Qi=\sum_{i=1}^{n}\frac{(Mi-Ii)}{(Si-Ii)},$$

where *Mi* the measured metal concentration for the ith sample (μg/l), *Ii* the ideal concentration for the ith parameter (I_i_ is 10, 3000, 10, 3, 50, and 2000 μg/l for As, Zn, Pb, Cd, Cr, and Cu, respectively), *Si* The standard value (highest permissible value for drinking water) for the ith parameter based on World Health Organization (WHO) guidelines (equal to 50, 5000, and 100 for As, Zn, and Pb, respectively).

A value of HPI below 100 indicates non-contaminated water, while a value above 100 suggests contamination by heavy metals. Furthermore, when the HPI reaches 100, it represents the threshold for dangerous contamination. The symbol (–) denotes the numerical difference between these two values, disregarding the algebraic sign^34,36^. While both the metal index (MI) and the heavy metal pollution index (HPI) serve to evaluate contamination levels, they do so through different approaches. MI directly measures contamination severity by assessing metal concentrations concerning their maximum allowable concentrations (MAC) values. This method is particularly adept at identifying metals that significantly exceed regulatory thresholds. On the other hand, HPI offers a more comprehensive assessment by factoring in metal toxicity through predetermined weighting factors derived from extensive scientific research and regulatory guidelines. This approach allows for a nuanced evaluation of pollution, considering each metal’s relative toxicity. In summary, MI excels at pinpointing metals that greatly surpass regulatory limits, while HPI provides a more holistic evaluation, making it effective in discerning metals with varying levels of toxicity and their contribution to overall pollution^33^.

#### Heavy metals evaluation index (HEI)

The HEI is a quantitative measure used to evaluate the levels of heavy metals in water samples. The HEI is calculated by summing the ratios of the measured concentration (Hc) to the maximum allowable concentration (Hmax) for each parameter. The measured concentration (Hc) is expressed in micrograms per litre (μg/l), while the maximum allowable concentration (H_max_) represents the threshold value set for each specific heavy metal. The HEI is calculated using the Eq. (8)^37^:

8 $${\text{HEI}} = \sum\limits_{{{\text{i}} = 1}}^{{\text{n}}} {\frac{{{\text{Hc}}}}{{{\text{H}}\max }}},$$

where *Hc* the measured concentration for the ith parameter (μg/l), *Hmax* the maximum allowable concentration for the ith parameter (μg/l).

An HEI value below 40 indicates a low level of heavy metal pollution, while an HEI value between 40 and 80 suggests a medium level of contamination. HEI values exceeding 80 indicate a high level of heavy metal pollution, which poses a significant risk to water quality and potentially to human health^37^. In comparing the HEI and the HPI, HEI offers a direct assessment by comparing measured concentrations to permissible limits. In contrast, HPI provides a more intricate evaluation, incorporating the relative toxicity of each metal through predefined weighting factors. HEI simplifies the evaluation of regulatory compliance, whereas HPI furnishes a nuanced appraisal of pollution, accounting for variations in metal toxicity. On the other hand, MI offers a direct measure of contamination severity, being particularly effective at identifying metals with high health risks. Nonetheless, it may not flag metals with lower concentrations that are still of concern. The choice between HEI and MI depends on the specific objectives of the assessment and the regulatory context^32^.

#### Contamination degree index (Cd)

The contamination degree index (Cd) is another index used for evaluating the degree of contamination caused by heavy metals in water samples. The Cd quantitatively measures the overall contamination level based on the concentrations of different heavy metals. The Cd is calculated using the equations (Eqs. 9, 10):

9 $$\text{Cd}=\sum \limits_{\text{i}=1}^{\text{n}}\text{Cfi},$$

10 $${\text{CFi}} = \frac{{{\text{CAi}}}}{{{\text{CNi}}}} - 1,$$

where *Cfi* contamination fit index for the ith parameter, *CAi* measured concentration for the ith parameter (μg/l), *CNi* the maximum allowable concentration for the ith parameter (As: 1, Cd: 3, Cr: 50, Cu: 2000, Fe: 300, Mn: 400, Ni: 20, Pb: 10, and Zn:5000 μg/l^38^.

The interpretation of Cd values depends on specific threshold levels or classifications established for different regions or regulatory bodies. Generally, higher Cd values indicate a higher degree of contamination, while lower Cd values suggest a lower level of contamination. Based on the obtained Cd values, water samples can be categorized into three levels: low contamination (Cd < 1), medium contamination (Cd = 1–3), and high contamination (Cd > 3)^24,39^. These categories provide further insight into the extent of heavy metal contamination in the water samples, helping to assess the potential risks associated with the measured concentrations.

In summary, Cd provides a comprehensive contamination assessment, considering multiple heavy metals. MI offers a straightforward evaluation, HEI directly assesses regulatory compliance, and HPI offers a comprehensive evaluation accounting for varying metal toxicity. The choice between these indices depends on the specific objectives of the assessment and the regulatory context.

### Local distribution and geo-statistical modelling

In this study, an assessment of the spatial distribution of water quality parameters was conducted in Shiraz’s drinking water distribution network. This analysis aimed to characterize the variations in water quality across the study area and generate zoning maps for the specified parameters. To determine the geographic coordinates of the sampling locations, a portable GPS device (Model No. GARMIN MONTANA 650, USA) was utilized, providing latitude, longitude, and Universal Transverse Mercator (UTM) coordinates^40^.

The collected sampling location coordinates were then imported into ArcGIS 10.4.1 software, a widely used geographic information system (GIS), to prepare zoning maps. Interpolation models were employed to effectively estimate and visualize the spatial distribution of water quality within the study area. Specifically, the inverse distance weighting (IDW) interpolation method was applied to create the zoning maps, allowing for a comprehensive understanding of the spatial variations in drinking water quality^41,42^.

The IDW method estimates values at unsampled locations based on known values from sampled locations. This process assigns higher influence to points closer to the unsampled location, emphasizing the principle that nearby measurements carry more weight in the interpolation. Moreover, the interpolation process entailed specific steps, including considering neighbourhood size and other pertinent parameters. These choices were made judiciously to ensure the accuracy and reliability of the spatial estimations. However, it is essential to acknowledge that, like any interpolation method, IDW has inherent limitations. These considerations include assumptions related to spatial autocorrelation and the potential sensitivity of results to parameter selections. Recognizing these aspects provides a well-rounded understanding of the methodology employed in evaluating the spatial distribution of water quality parameters in this study^43^.

### Uncertainty analysis by Monte Carlo simulation (MCS)

Human health is often subject to uncertainties, which, if not properly addressed, can result in the loss of valuable information. Therefore, it leads to ineffective decisions, far from reality, or inaccurate about protecting human health. Uncertainty analysis is crucial in assessing modelling results’ reliability and robustness. This study employed Monte Carlo simulation (MCS) as a powerful technique for uncertainty analysis in water quality assessment^18^.

Monte Carlo simulation is a statistical method that uses random sampling to explore the uncertainty associated with input parameters and their impact on the model’s output. By generating many random samples within specified parameter ranges, MCS allows for assessing the variability and distribution of model outputs, providing insights into the range of possible outcomes and associated uncertainties. Measured heavy metal concentrations, ingestion rate, body weight, and duration of exposure were used to determine the distribution of potential uncertainty. The calculations were repeated 10,000 times, and finally, the results are indicated with a confidence level in the 1–99% range. Through the iterative process of generating multiple simulations, each with different input parameter values, MCS estimates probability distributions for model outputs. This information can then be used to assess the likelihood of specific water quality scenarios, identify sources of uncertainty, and inform decision-making processes related to water management and risk assessment^18^.

In this study, employing the R software environment, we conducted rigorous Monte Carlo simulations and sensitivity analyses to comprehensively assess non-carcinogenic and carcinogenic risks. The initial step involved determining distribution functions for each elemental parameter using established R packages, including fitdistrplus, logspline, EnviroPRA, and survival^44^. Specifically, we modelled the parameters for Arsenic (As), Chromium (Cr), Cadmium (Cd), Lead (Pb), Zinc (Zn), Copper (Cu), and Manganese (Mn) utilizing the following distributions shown in Table 3.

**Table 3:** Distribution functions and parameters for MCS.

Parameters		Distribution function	Values	Ref.
As		Log-normal	[0.212 ± 0.896]*	^45^
Cr		Gamma	[2.436 ± 0.125]	
Cd		Normal	[0.071 ± 0.060]	
Pb		Normal	[3.45 ± 2.61]	
Zn		Log-normal	[3.478 ± 1.295]	
Cu		Normal	(3.509 ± 2.27)	
Mn		Weibull	[2.207 ± 52.088]	^46^
BW	Children	Normal	[15, 3]	^47^
	Adults	Normal	[70, 7]	
IR	Children	Normal	[1, 0.2]	
	Adults	Normal	[2, 5]	

*Mean ± SD.

## Results and discussion

### Heavy metals concentration

Heavy metals’ concentration and distribution in groundwater resources depend on mineral composition, soil compound and underground stones and their geological properties, hydro-chemical features, and anthropogenic activities on the ground surface^48^. Table 4 presents the summary statistics of pollutant concentrations in the collected samples and the potable water standard specifications provided by the WHO and EPA.

**Table 4:** Summary statistics of pollutant concentration in samples and potable water standard specifications given by the WHO.

Heavy metals	Wet season (g/l)			Samples exceeding DW. standards (%)	Dry season (g/l)			Samples exceeding DW. standards (%)	WHO standard (µg/l)	EPA standard (µg/l)
	Min	Max	Mean		Min	Max	Mean			
As	0.3	2.44	2.23	0	0.33	8.8	1.6	0	10	10
Cd	0	10	0.31	2.4	0	9	0.29	2.4	3	5
Pb	0	9.89	2.66	0	0.67	9.89	4.22	0	10	15
Cr	1.6	42	24.99	0	0.75	24	13.97	0	50	100
Cu	1.12	560.33	4.54	0	0	96	11.35	0	2000	1300
Zn	4.31	368.83	69.7	0	5.8	730	140.62	0	–	500
Mn	4	163	44.14	0	13	92	97.29	0	400	500

As shown in Table 4, the maximum mean concentration of heavy metals in groundwater was as follows: Zn > Mn > Cr > Cu > pb > As > Cd. The mean concentrations of As, Cd, Pb, Cr, Cu, Zn, and Mn during the study period were lower than the standard limitations determined by WHO^20,49^.

### Groundwater contamination and the distribution of heavy metals concentrations

**Figure 2:**
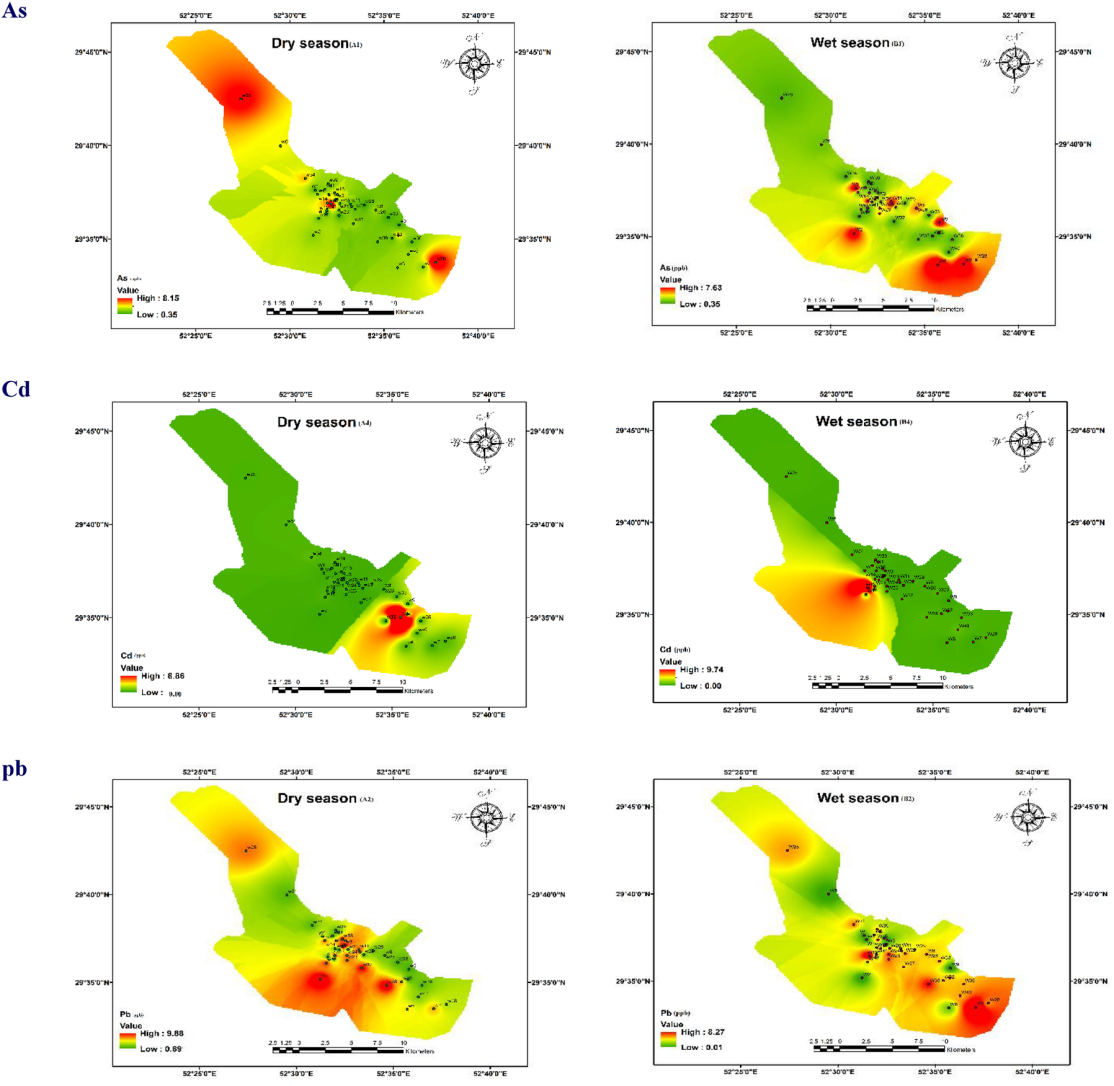

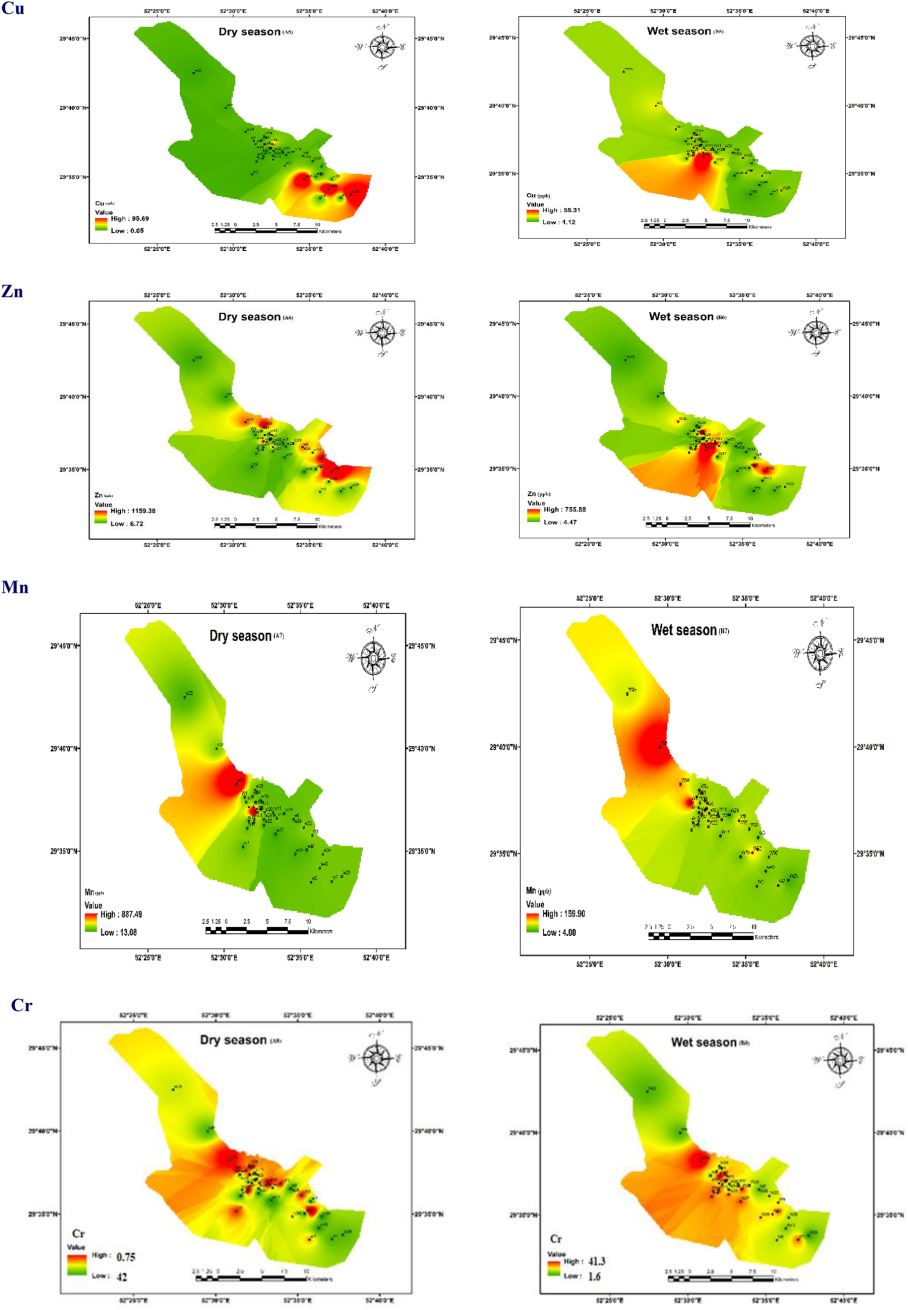
Geographical distribution of studied metals concentration in wet and dry seasons.

The zoning maps of all heavy metals concentration in two wet and dry seasons are given in Fig. 2. Also, the results from Table 4 indicate the summary statistics of heavy metal concentrations in the collected groundwater samples and compare them with potable water standards recommended by the World Health Organization (WHO) and the Environmental Protection Agency (EPA). For As, the mean concentrations in wet and dry seasons were 1.6 ± 1.13 and 2.29 ± 2.02 μg/l, respectively, and a meaningful statistical difference was observed between the measured concentrations in the two seasons (p < 0.05). The maximum permissible concentration of arsenic is ten μg/l in drinking water (Iran, E.P.A., and WHO standards)^10,50^. Therefore, the mean As concentration for 95% of samples was within the permissible limit provided by the mentioned organizations^42,51^. As Fig. 2 shows, the maximum concentration was observed in the northern area, wells 25, 28, and 40, in the dry season, and the lowest concentration was related to wells number 33 and 38 with values of 8.28 and 0.35 μg/l, respectively.

Moreover, the highest concentrations were in the wet season measured in wells placed in south and southeast regions, and the lowest value was related to sampling well 15 in the north with the value of 0.53 μg/l. During the study, an arsenic concentration increase was observed from the north and northwestern to central regions, predominantly south and southeastern parts of the research area. So, the water quality decreased during this time. It can be due to the hydraulic slope of the Shiraz aquifer (from north to south). Also, the arsenic concentration is influenced by human-made contamination, ion leaching, direct wastewater discharge, and natural processes such as dissolution and penetration in the studied area^52,53^.

Cadmium (Cd) concentrations remained within acceptable limits during both seasons. The mean concentration of cadmium in both low-rainfall and high-rainfall seasons were 0.29 + 1.39 μg/l and 0.31 ± 1.3 μg/l, respectively, and there is no statistical difference between the two seasons’ concentrations. Only 2.4% of samples had higher values than the maximum recommended value of Iran, WHO, and EPA recommendations^7^. According to Fig. 2, the trend of Cd concentration was changed from the southern area in the dry season to the south and southwestern regions of the study area in the wet season (max = 8.86 µg/l in the wet season and 8.17 µg/l in the dry season), with a broader range of the study area experiencing water quality deterioration over time. The high levels in central, southern, and southwestern areas may be due to oxidation and acidification through groundwater pumping, excessive nitrate entry through agricultural fertilizers, and even industrial zones west of the study area^40^.

Lead (Pb) concentrations showed no exceedances of standards during the dry and wet seasons**.** The mean concentrations of lead in cold and warm seasons were 2.66 and 4.22 μg/l, respectively, and there is no statistical difference in both seasons. All sample concentrations were over the allowable range (WHO and EPA standards of 10 μg/l). The maximum concentration was observed in southwestern and western regions, wells number 2 and 38, in the dry season with the values of 9.88 and 9.5 μg/l, respectively. In the wet season, the concentration trend was changed to the southeastern region, and well number 7 had the highest concentration. The intensive presence of lead in these regions can be related to the mixed effect of human and natural resources, such as anthropogenic activities like the application of fertilizers and pesticides containing Pb (e.g., Aldrin, Dieldrin, and endosulfan) in agricultural lands, which is resulted in the high concentration of lead in groundwater^54^. In fertilizers, these heavy metals can be leached from the soil and penetrate groundwater^55^.

Copper (Cu) concentrations showed no exceedances of the EPA standard of 1300 µg/l, but during the wet season, some samples had concentrations as high as 560.33 µg/l, indicating potential localized contamination. Cu can be found in human tissues and is essential in making Red Blood Cells (RBC) and protecting neurons and the immune system^20^. The mean Cu concentrations in low- and high-rainfall seasons were 4.28 ± 3.39 and 4.43 ± 5.3 μg/l, respectively, without any statistical difference. All samples meet Iranian drinking water quality guidelines (number 1053) and EPA and WHO water quality standards, which had values below 2000 μg/l. The maximum Cu concentration was related to well numbers 12 and 23 in the dry and wet seasons. Therefore, changes in Cu concentration were detected from the southeastern south and southwestern regions. Cu concentration in groundwater resources is primarily influenced by the long-term interactions between water and rocks and the redox environment of the groundwater system^56^.

Zn is necessary for good performance, immune system health, metabolic activities, proper DNA synthesis, healthy growth, and wound healing. In contrast, its deficiency leads to delayed growth and makes the person susceptible to disease^57^. There was no statistical difference between valued concentrations wet (ranged from 5.8 to 730 μg/l, mean = 460.62 μg/l) and dry (varied from 4.31 to 368.83 μg/l, mean = 69.7 μg/l). According to Fig. 2, the maximum levels were moved from east and southeast to south and southwest of the study area in low-and high-rainfall seasons, respectively. The presence of Zinc in phosphate and urea fertilizers indicates that agricultural activities can be considered the primary human sources of groundwater resources. Zn may be washed and leached from soil to groundwater resources^55^. Also, the concentration is primarily affected by the long-term interaction of water, rocks, and the redox environment of the groundwater system^58^.

The Mn measured values in groundwater samples varied from 4 to 163 μg/l (mean = 44.14 μg/l) in the wet season and ranged from 13 to 92 μg/l (mean = 97.29 μg/l) in the dry season with no statistical difference. Based on the geographical distribution map, the maximum concentration was quantified in northern, central, and western regions in both seasons. The content of Mn in the tailings is very high. The tailings are oxidized during long-term stacking to produce a large amount of acid, which promotes the dissolution of Mn-containing minerals and increases the Mn content^59^.

### Evaluation of pollution indices and the toxic parameters

**Figure 3:**
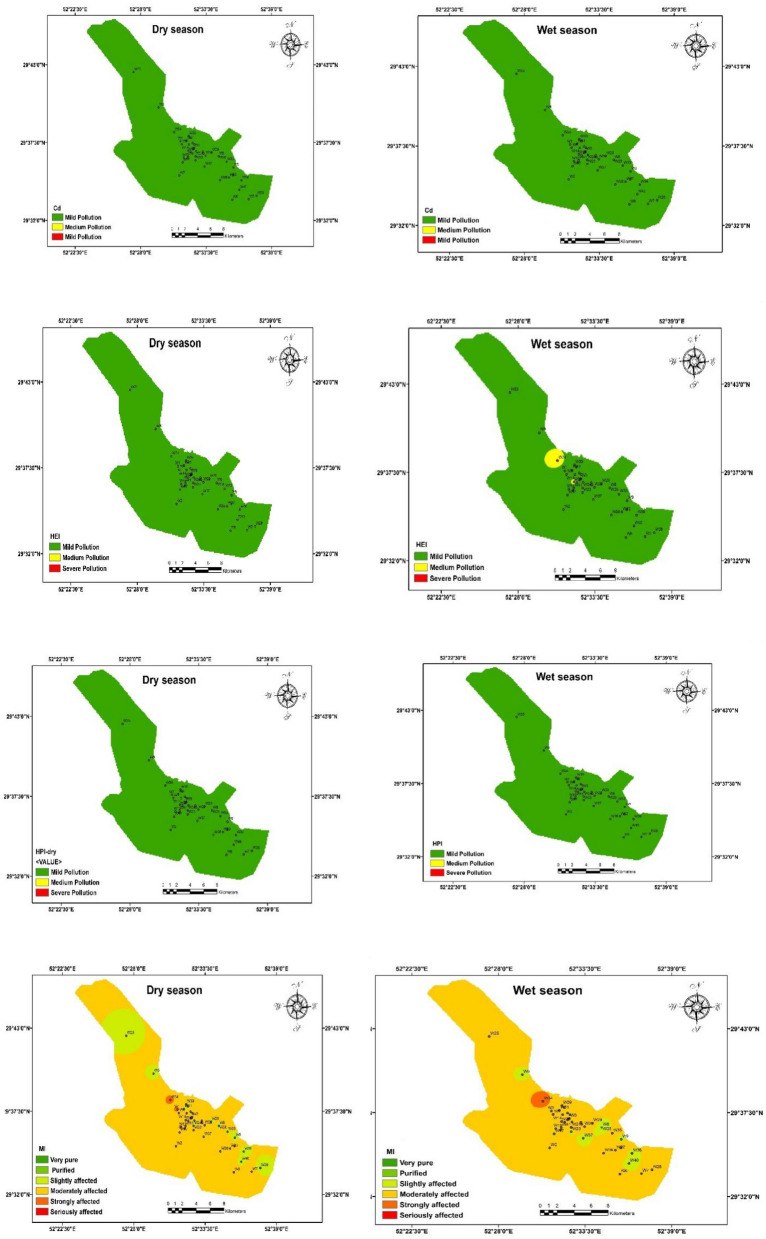
The classification values map of (Cd), (HEI), (HPI), and (MI) in dry and wet seasons.

The studied metals concentration must be compared with their maximum permissible limit in standard mode to calculate the metal index and determine the water resources pollution degree to heavy metals. Figure 3 shows the values of studied indexes for all samples. The results depict that Cd has been a cumulative index evaluated as the sum of the pollution factor index for studied metals in both seasons. This index compares the measured metal concentrations with each metal’s highest permissible concentration limit^38^. Based on the results, Cd ranged from − 2.96 to 16.37 (mean = 2) and − 1.64 to 36.5 (mean = 7.3) in the wet and dry seasons, respectively. During the wet season, Cd of groundwater in 25%, 7.5%, and 67.5% of the regions shows high, medium, and low contamination. Similarly, during the dry season (Fig. 3), Cd of groundwater in 40% of the samples indicates a heavy contamination degree at over 3; Cd of groundwater in 2% of the were in the medium degree contamination range; and Cd in the remaining 55% of all the samples were classified in the low degree.

Also, HEI varied from 3.30 to 12.74 (mean = 5.03) in the dry season and varied from 3.91 to 31.23 (mean = 6.97) in the wet season. The HEI of groundwater in 2.5% and 97.5% of the studied areas shows medium and low contamination, respectively, in both seasons. Values evaluation results of the HPI model show that during the wet and dry seasons (Fig. 3), the HPI evaluation value of the groundwater in all sampling areas is within the safe limit at less than 50. The heavy metals index (MI) is used to determine the effect of heavy metals on human health. Evaluation results indicated that the MI values in 15% and 30% of the studied are non-drinkable in dry and wet seasons, respectively. Moreover, MI in the remaining regions’ samples is within the drinkable and threshold classification. The point is that in this index, if the value of only one of the metals exceeds the maximum permissible limit, the index value becomes more than one and is placed in the non-drinkable class^32^.

Many types of research are conducted to evaluate water quality using various indexes worldwide. Jahromi et al. assessed the groundwater resource’s drinkability quality in Varamin’s aquifer. Severe changes in the metal concentration were observed, and the aquifer pollution was not dangerous regarding heavy metals. Jafari and Hassan Zadeh^60^ investigated the water quality of Anzali Wetland for heavy metals using the HPI. The findings of the HPI model showed moderate heavy metal contamination and severe pollution in the eastern part of Anzali Wetland. The results of Nasr Abadi’s research^13^ showed that the mean values of Cd and HPI were significantly lower than the danger threshold.

### Risk assessment

#### Non-carcinogenic

**Figure 4:**
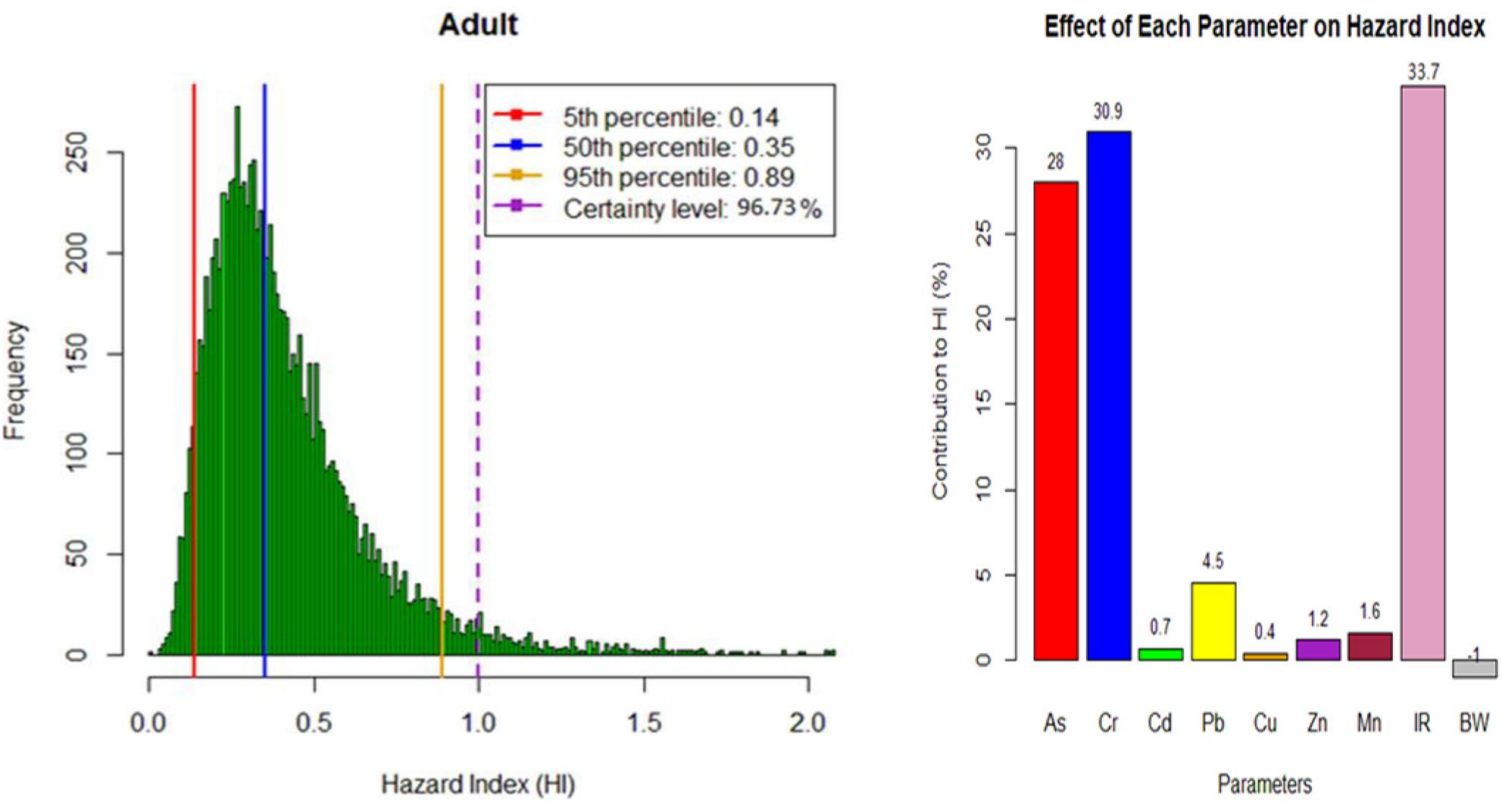
Histograms and sensitivity analysis of hazard index (HI) in heavy metals for children and adult groups.

This study assessed the non-carcinogenic risk of heavy metals in a residential area’s drinking groundwater using deterministic and probabilistic methods. Table 5 and Fig. 4 provide a summary of the non-carcinogenic risk distribution for selected heavy metals, including Arsenic (As), Chromium (Cr), Cadmium (Cd), Lead (Pb), Zinc (Zn), Copper (Cu), and Manganese (Mn), for two different exposure groups: children and adults. The risk assessment was based on each metal’s chronic daily intake (CDI) values and hazard quotient (HQ)^61^. The calculations were based on the average of two seasons. Overall, the mean non-carcinogenic risk of As, Pb, Cr, and Mn in children is higher than in adults.

**Table 5:** Non-carcinogenic risk distribution from heavy metals in the studied drinking groundwater.

Metals	Children		Adult	
	CDI	HQ	CDI	HQ
As	0.1278	0.426	0.0548	0.183
Cr	1.223	0.408	0.524	0.175
Cd	0.005	0.009	0.002	0.004
Pb	0.23	0.066	0.099	0.028
Zn	5.069	0.017	2.172	0.007
Cu	0.234	0.006	0.1	0.003
Mn	3.07	0.022	1.316	0.009

HI				
	Children		Adult	
Min	0.461		0.089	
Mean	1.260		0.402	
Max	2.850		1.071

Table 5 and Fig. 4 show that the non-carcinogenic risk levels vary among the studied heavy metals and the different age groups. Among the studied metals, Arsenic (As) and Chromium (Cr) have relatively higher non-carcinogenic risk levels than other metals. The CDI values for As in children and adults are 0.1278 and 0.0548 mg/kg/day, respectively, with corresponding HQ values of 0.426 and 0.183. Similarly, the CDI values for Cr are 1.223 and 0.524 mg/kg/day for children and adults, respectively, with HQ values of 0.408 and 0.175. However, even for these metals, the HQ values remain below 1, indicating that the health risks associated with their exposure are still within safe limits for both age groups. However, it is important to note that long-term exposure to heavy metals, even at low levels, can still have cumulative effects on health over time. Hence, continuous monitoring and assessment of water quality are essential to ensure public health safety^62^.

The hazard index (HI) values presented in the table signify the non-carcinogenic health risks associated with the examined heavy metals in the drinking groundwater, applicable to both children and adults. The HI values measure the cumulative health risk from exposure to multiple heavy metals. For the children’s group, the HI values range from a minimum of 0.461 to a maximum of 2.850, with a mean value of 1.260. These HI values suggest that, on average, children may be exposed to a health risk above the safety threshold of one, indicating a potential concern for adverse health effects from heavy metal exposure^5^.

In contrast, for the adults’ group, the HI values range from a minimum of 0.089 to a maximum of 1.071, with a mean value of 0.402. The mean HI value below one indicates that adults’ overall non-carcinogenic health risk is within acceptable limits, suggesting a relatively lower potential health risk than for children. The variations in HI values between children and adults can be attributed to differences in sensitivity to heavy metal exposure and water consumption patterns between the two age groups^63^.

In addition, the MCS technique, conducted through coding in R software version 4.2.2, considered the variability and uncertainty in input parameters such as contaminant concentration, ingestion rate, and body weight. This approach allowed for a comprehensive evaluation of potential risks associated with heavy metal exposure in different age groups. The histograms depicting the probabilistic approaches for heavy metal concentrations in the exposed groups were presented in Fig. 4 for children and adults. Sensitivity analyses were conducted to assess the influential factors on risk assessment, considering the sensitivity of various parameters. By systematically varying the input parameters in the Monte Carlo simulation, the sensitivity analysis allowed us to identify the key contributors to the variability in health risk estimates associated with heavy metal exposure. These findings provide valuable insights into which parameters significantly impact the overall risk assessment, aiding in prioritizing control measures and mitigation strategies to safeguard public health^63^. The simulation results showed that HI Values for the 95th percentile in the children and adult age groups were 5.28 and 0.89, respectively, indicating a non-carcinogenic risk for children groups. High-risk levels in infants can be due to their low body weight compared to other age groups. Also, the difference in HI values between the children and adult groups can be attributed to the probabilistic nature of the MCS method. Unlike the deterministic method used to calculate Hazard Quotient (HQ), which relies on fixed values and assumptions, the MCS accounts for uncertainties and variations in exposure and toxicity data. In the case of children, the variability and uncertainty in factors such as contaminant concentrations, ingestion rates, and body weights may lead to a more comprehensive range of possible outcomes in the MCS simulation. Consequently, this broader distribution of HI values for children includes higher values, indicating the possibility of increased non-carcinogenic health risks^20^.

On the other hand, the deterministic method for calculating HQ might have provided a single value that falls below the threshold of concern (HQ < 1), potentially underestimating the true range of potential risks. Therefore, the MCS approach offers a more comprehensive and realistic assessment of non-carcinogenic risks, capturing the uncertainty and variability inherent in the data and providing a more accurate representation of the health risk profile for heavy metal exposure, especially in vulnerable populations like children^6^.

#### Carcinogenic risk assessment

Table 6 presents the results of the carcinogenic risk assessment associated with three specific heavy metals (Arsenic, Chromium, and Cadmium) in the drinking groundwater, categorized by different age groups (Children and Adults). The evaluation was conducted based on cancer risk parameters to understand the potential health implications of exposure to these metals.

**Table 6:** Carcinogenic risk of heavy metals in drinking groundwater by age groups.

Groups	Parameter	As	Cd	Cr
Children	Min	3.38571E−06	0.00E +00	6.84E−06
	Mean	1.64289E−05	1.54E−07	1.99E−05
	Max	4.03714E−05	3.8E−07	3.31E−05
Adult	Min	1.69E−05	0.00E+00	3.42E−05
	Mean	8.21E−05	7.71E−07	9.96E−05
	Max	2.02E−04	1.9E−06	1.66E−04

	TCR			
	Children		Adult	
Min	1.02E−05		5.11E−05	
Mean	3.65E−05		1.82E−04	
Max	7.39E−05		3.69E−04	

The values in Table 6 represent the estimated excess lifetime cancer risk (ELCR) associated with each heavy metal for the different age groups. ELCR values are expressed in terms of risk per million individuals and provide insights into the likelihood of cancer development due to long-term exposure. Analyzing the results, we observe that the ELCR values for all heavy metals and age groups are generally quite low, indicating a relatively low potential for cancer risk through exposure to these metals in the drinking groundwater. The calculated ELCR values range from as low as 0 (no risk) to a maximum of around 4.56E−04, corresponding to a very low fraction of the population potentially developing cancer due to heavy metal exposure^64^.

Additionally, the values follow a consistent pattern, with Children generally having slightly lower ELCR values than Adults. This can be attributed to the fact that Children, being more susceptible due to their developing physiology, tend to have slightly higher exposure levels. Despite this trend, all values remain well below the acceptable cancer risk threshold, typically set at 1E−06 (or 1 in a million)^6^.

The absence of significant carcinogenic risk despite the presence of non-carcinogenic risk, as indicated by the results in Tables 5 and 6, could be attributed to the different assessment approaches for these two types of risks. The differing outcomes between non-carcinogenic and carcinogenic risk assessments can be attributed to the differences in the toxicological properties of these heavy metals, the specific exposure pathways, and the calculated parameters used for each assessment method^65^. The absence of significant cancer risk despite non-carcinogenic risk could indicate that while exposure to these heavy metals might pose some non-carcinogenic health risks, the probability of developing cancer due to this exposure is minimal^5^.

**Figure 5:**
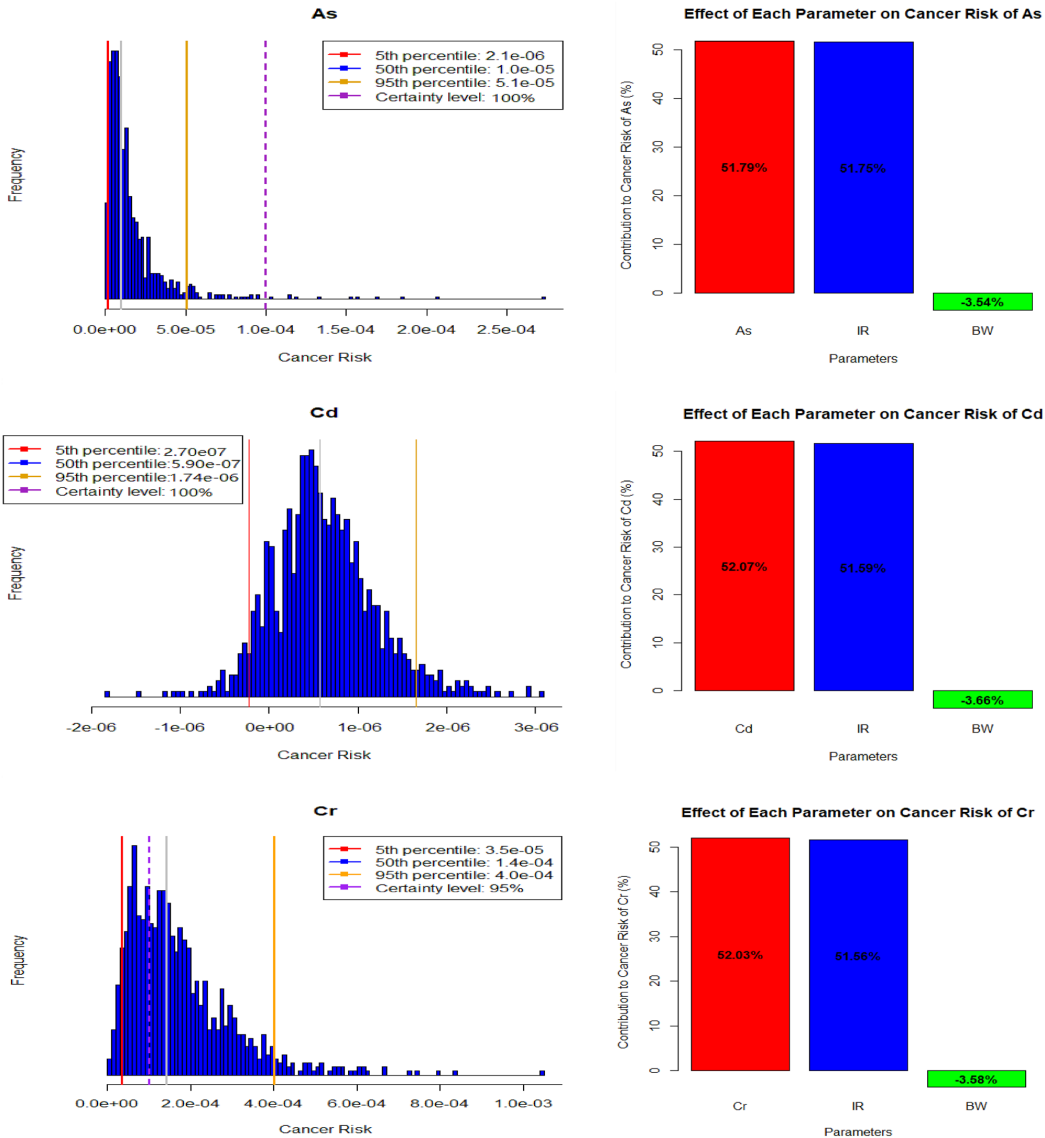
Histograms of the uncertainty analysis and sensitivity analysis of children’s group.

**Figure 6:**
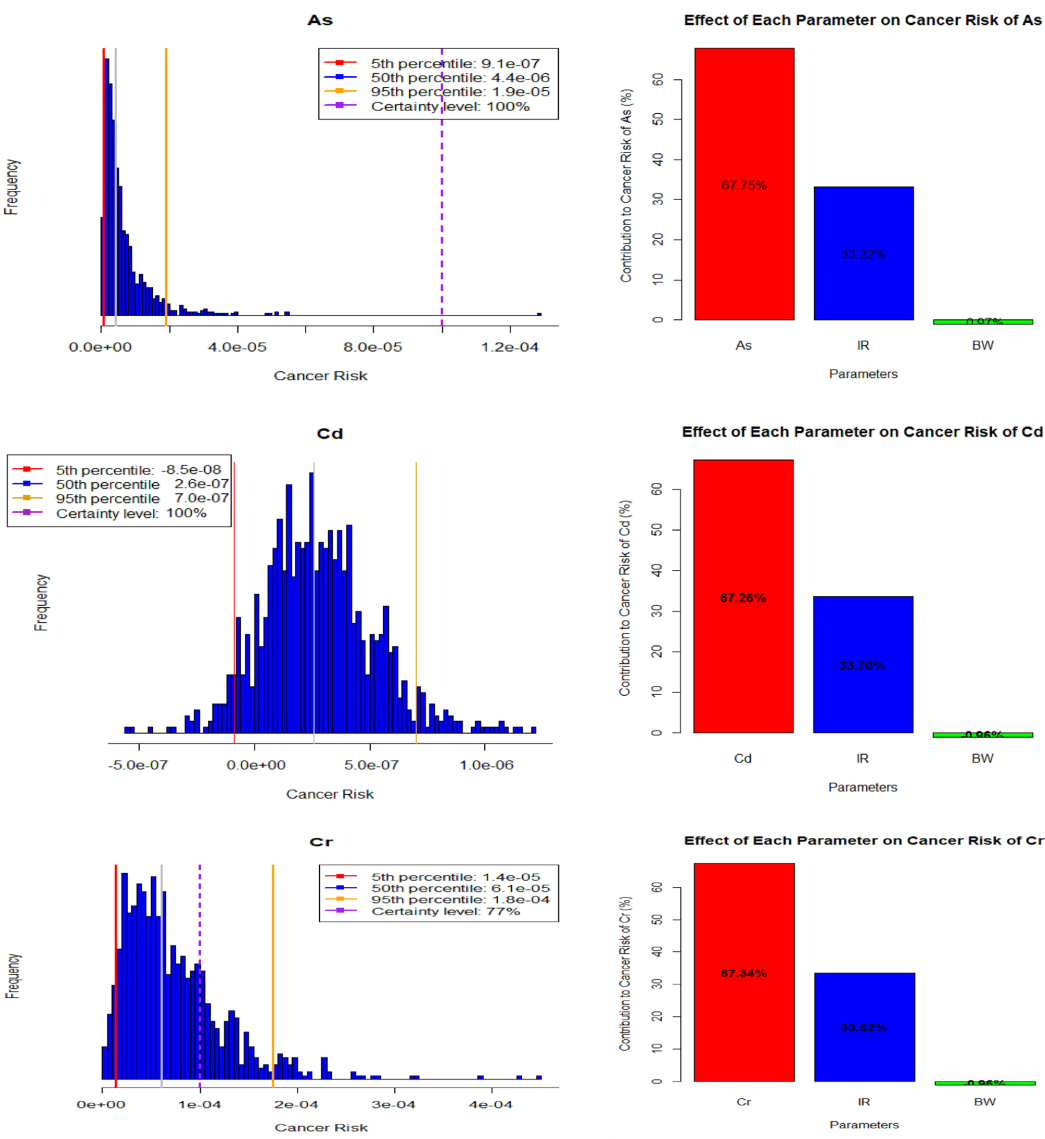
Histograms of the uncertainty analysis and sensitivity analysis of the adult group.

In conjunction with the quantitative data, we have utilized histograms and diagrams for sensitivity analysis to visually expound upon the dimensions of uncertainty and sensitivity within the context of carcinogenic risk assessment (Figs. 5, 6). The histograms offer a graphical dissection of risk level distribution across discrete intervals, while the sensitivity analysis diagrams shed light on the influences of discrete parameters on the calculated risk values.

These Figs. 5 and 6 reveal percentile values representing different risk levels for Arsenic (As), Cadmium (Cd), and Chromium (Cr). Specifically, for arsenic in the children group, the percentiles (5th, 50th, and 95th) of 2.1E−06, 1.0E−05, and 5.1E−05, respectively, denote the varying potential risk levels to which individuals in the children group could be exposed. However, it is essential to note that these values alone do not directly convey health impacts. The significance of these values is best understood when compared to established health standards or guidelines^57^.

On the other hand, the sensitivity analysis provides insights into the influence of specific parameters on the overall carcinogenic risk assessment. In this case, As concentration, Ingestion rate (IR), and body weight are identified as contributing factors. Arsenic concentration and Ingestion rate contribute by 51.79% and 51.75%, respectively, indicating their strong influence. Notably, Body weight, with a contribution of − 3.54%, appears to have a minor inverse impact. Similar trends for Cd and Cr are observed, with varying percentiles and corresponding sensitivity analysis outcomes. It is important to remember that interpreting these values regarding health impacts necessitates referencing relevant health guidelines. These indicators guide further assessment and informed decision-making regarding potential health risks associated with heavy metal exposure^66^.

The insights drawn from the adult group’s analysis (Fig. 6) echo the patterns observed in the children group, albeit with distinct percentile values. The percentiles for As, ranging from 9.1E−07 to 1.9E−05, denote potential risk levels. The sensitivity analysis for As, Cd, and Cr mirrors the findings in the children group, reaffirming the significant impact of parameters like concentration and ingestion rate. The contributions of As concentration and Ingestion rate, which are 67.75% and 33.22%, respectively, highlight their prominent influence on the overall carcinogenic risk assessment. Conversely, Body weight, contributing by − 0.97%, exhibits a relatively minor inverse effect, consistent with the trends identified in the children group^6^.

This alignment in trends underscores the robustness of the results across different age groups and provides a comprehensive understanding of the potential health risks associated with heavy metal exposure. The percentiles and sensitivity analysis offer valuable insights that guide further evaluation and decision-making processes related to health risk management and prevention strategies.

## Conclusion

This cross-sectional study analysed 80 water samples from 40 designated stations in Shiraz, Iran, for heavy metal contamination across wet and dry seasons. We comprehensively assessed contamination risks by employing advanced Monte Carlo simulations driven by R software. The heavy metals index (MI) and heavy metals evaluation index (HEI) were pivotal in quantifying contamination levels and associated health risks. Our approach heightened precision in non-carcinogenic and carcinogenic risk assessments, providing critical insights into the complex interplay of heavy metal pollution, groundwater quality, and human health. This research significantly informs risk management strategies. Future studies may explore the long-term effects of heavy metal exposure on diverse demographics and consider the cumulative impact of multiple heavy metals on health.

## Data Availability

The data generated and analyzed during this study are available within the study.
